# The effect of *Limosilactobacillus fermentum* 2i3 and 0.6% addition of humic substances on production parameters and the immune system of broilers

**DOI:** 10.1016/j.psj.2024.103884

**Published:** 2024-05-22

**Authors:** E. Hudec, D. Mudroňová, S. Marcinčák, M. Bartkovský, A. Makiš, M. Faldyna, M. Ratvaj, V. Karaffová

**Affiliations:** ⁎ Department of Morphological Disciplines, University of Veterinary Medicine and Pharmacy, Košice, Slovakia; † Department of Microbiology and Immunology, University of Veterinary Medicine and Pharmacy, Košice, Slovakia; ‡ Department of Food Hygiene and Technology, University of Veterinary Medicine and Pharmacy, Košice, Slovakia; § Veterinary Research Institute, Brno, Czech Republic

**Keywords:** immune parameter, *limosilactobacillus*, humic substance, broiler chicken

## Abstract

The widespread use of antibiotics in the poultry industry as growth promoters has led to the emergence of bacterial resistance, which poses a significant health risk to humans and animals. Substances of natural origin, such as probiotic bacteria and humic substances, can be a promising solution. The aim of this experiment was to study the effect of the administration of a probiotic strain of *Limosilactobacillus fermentum* 2i3 and/or a new formula of humic substances specifically designed for detoxification on the production parameters, including gene expression of myogenic growth factors and selected parameters of the immune response. We found that production parameters such as feed conversion ratio and weekly weight gain, as well as gene expression of mucin-2 and immunoglobulin A, were positively influenced mainly by the administration of *L. fermentum* 2i3. Similarly, the percentage of active phagocytes and their absorption capacity as well as the proportions of CD8+ and CD4+CD8+ T-lymphocyte subpopulations were significantly increased. The addition of humic substances, either alone or in combination with probiotics, significantly reduced the aforementioned parameters compared to the control. On the other hand, the relative gene expression for all myogenic growth factors was the highest in the humic group alone. Based on the results obtained, we can confirm the immunostimulating effect of *L. fermentum* 2i3 administered in drinking water, which also had an impact on important production parameters of broiler meat. On the other hand, in the combined group there was no expected potentiation of the positive effects on the observed parameters.

## INTRODUCTION

The production of livestock products such as poultry meat and eggs has seen a growing trend in Europe in recent years (De Cesare et al. 2022; Kralik et al. 2018). The increase in popularity of consumption of chicken meat is mainly due to its low price, as well as its many nutritional values, including biologically available high-quality proteins, fats, low cholesterol, and fiber. Moreover, chicken meat is also a good source of selenium and provides zinc, copper, phosphorus, magnesium and iron (Choi et al. 2023).

Thanks to the ban on the use of antimicrobial substances as growth promoters in broiler nutrition (López-García et al. 2023), wide range of possibilities is opened for the use of substance of natural origin such as probiotic bacteria, prebiotics, synbiotics, postbiotics, humic substances, essential oils, herbal extracts, essential oils, organic acids, enzymes and essential amino acids (Abd El-Hack et al. 2022; Phillips et al. 2023).

Probiotic bacteria are undoubtedly the most studied. *Limosilactobacillus* spp., *Enterococcus faecium, Bifidobacterium* spp. and others are among the lactic acid bacteria (**LAB**) used in various probiotic application forms. They are widely preferred for their beneficial effects, including maintaining a healthy balance of bacteria in the gut, improvement of digestion and metabolic functions, and last but not least, modulation of the immune system (Ramlucken et al. 2020; Shehata et al., 2022; Zhang et al. 2022).

Humic substances (**HS**) are natural organic compounds formed by the biological and chemical decomposition of plant biomass via the activity of microorganisms. HS can be extracted from leonardite and lignites or from environmental sources like compost and worm compost (Akaichi et al. 2022). In general, humic substances have antibacterial, antiviral, antithyroid, anti-inflammatory effects, improve immunity as well as reduce mortality in various animal species (Skalická et al., 2022; Vašková et al., 2023). On the other hand, the effect of humic substances in different concentrations, as well as their combination with other substances of natural origin, represents a so far insufficiently researched area with a high potential for use in practice. Therefore, our aim in this experiment was to study the effect of administration of a probiotic strain of *L. fermentum* 2i3 and/or a new formula of humic substances originating from leonardite in 0.6% concentration on the production parameters (chicken weight, average daily gain, feed consumption, feed conversion) including gene expression of growth factors (IGF-2, PAX-7, MYF-5) and selected parameters of immune response (IgA, MUC2, phagocyte function, lymphocyte phenotyping).

## MATERIAL AND METHODS

### Preparation of Probiotic Strain

The probiotic strain *Lactobacillus fermentum* 2I3 (new nomeclature: *Limosilactobacillus fermentum*) was isolated from the intestinal contents of a healthy domestic pheasant at the Department of Microbiology and Immunology UVLF in Košice. The strain was tested in several in vitro and in vivo experiments, which confirmed its probiotic properties (Koščová et al. 2006; Mudroňová et al. 2006; Englerová et al., 2018; Harčárová et al., 2021). The strain *L. fermentum* 2I3 was cultured in MRS broth (Merck, Germany) for 18 h at 37°C in a shaking water bath. A fresh 18-h culture was centrifuged for 15 min at 900 x *g*. The sediment was resuspended in the tap water and centrifuged once more. The washed sediment was again resuspended in the tap water so that the resulting concentration of bacteria was 10^9^ in 1 mL of water that was fed to the chickens.

### Experimental Scheme

The experiment was carried out in accordance with the European Parliament and Council Directive 2010/63/EU on the protection of animals used for scientific purposes and the animal protocol approved by the Ethical Committee for Animal Care at the University of Veterinary Medicine and Pharmacy in Košice (EKVP/2022-08). All recommended requirements for feeding, nutrition and welfare of chickens were followed.

For the trial, 160 one-day-old Ross 308 (hybrid meat chicken) broiler chickens purchased from a supplier (Hydina Slovensko, Ltd., Slovak Republic) were randomly divided into one control group (C) and 3 experimental groups, each group consisting of 40 pieces. All chickens in the control and experimental groups were fed: a starter diet (BR1) during the first 10 d of fattening; a growing diet (BR2) from d 11 to 28; a final diet (BR3) from d 29 to 35. The composition of the feed mixtures used (BR1, BR2, BR3; DeHeus, Bučovice, Czech Republic) is shown in Table 1. The control group of animals was fed with basic feed mixtures (starter, grower, and finisher) without supplementation with HS or probiotics. Chickens of the first experimental group (H) were fed with a diet enriched with 0.6% supplementation of HS (HUMAC® Natur AFM MycotoxiSorb, Humac s.r.o., Košice, Slovakia) (Table 2) from the second day, and the amount of the feed mixture was reduced by the amount of HS added. Chickens of the second experimental group (P) were supplemented with 1 mL of probiotic strain *L. fermentum* 2I3 (10^9^ CFU/mL) per chicken and per day from d 1. Probiotics were administered to chickens in the drinking water. Chickens of the third experimental group (PH) were fed with a combination of the previously described HS and probiotics with the same application schedule. All recommended requirements for feeding, nutrition, and welfare of chickens were followed.

**Table 1:** Composition of commercial feed mixtures for broilers.

A component of the feed mixture	BR1	BR2	BR3
Proteins (g)	221	186	180
Lipids (g)	38	34	43
Fiber (g)	36	36	37
Ashes (g)	90	60	100
Methionine (g)	5,6	5,3	4,9
Vitamin A (IU)	10800	10800	8100
Vitamin D3 (IU)	2400	2400	1800
Vitamín E (mg)	36	42	37
Copper (mg)	12,3	12	11
Salinomycin sodium (mg)	70	70	0

**Table 2:** Humac natur AFM Mycotoxisorb composition.

Feed ingredients	Content
Humic acids in dry matter	min. 65 %
Free humic acids in dry matter	min. 60 %
**Other substances in dry matter**	
Fulvonic acids	min. 5%
Calcium (Ca)	42 278 mg/kg
Magnesium (Mg)	5 111 mg/kg
Iron (Fe)	19 046 mg/kg
Copper (Cu)	15 mg/kg
Zinc (Zn)	37 mg/kg
Manganese (Mn)	142 mg/kg
Cobalt (Co)	1,24 mg/kg
Selenium (Se)	1,67 mg/kg
Vanadium (V)	42,1 mg/kg
Molybdenum (Mo)	2,7 mg/kg
All naturally occurring trace elements	in μg/kg
**Features**	
Particle size	up to 100 μm
Humidity	max. 15 %

Source: www.humac-farma.sk.

Broilers were reared on deep litter under controlled conditions. The animals had access to water and feed ad libitum during fattening. Feed and drinking water consumption will be monitored separately for each group of chickens until the end of the experiments (38–39 d). The body weight of individual broilers and feed consumption in chicks was measured at weekly intervals. The feed consumption was recorded each day, and the feed conversion ratio was calculated at the end of the experiment. The carcass yield was determined as a proportion of the body weight before slaughter and after evisceration.

### Production Parameters

#### Determination of Chicken Weight and Average Daily Gain

The body weight of the individual broilers determined by weighing all animals in each group on the first day of fattening was measured at weekly intervals (1, 2, 3, 4, 5 and the day of slaughter). After finishing fattening, the average body weight and the average daily gain of the broilers were calculated. The average daily gain was calculated as the weight calculated on the basis of the weight difference of 2 consecutive weighings over a certain period. Based on these results, the average daily gain “ADG” was subsequently calculated according to the following formula:

$$ADG\equiv \frac{H1-H2}{n}$$

H_1_ = the weight on the day of weighing; H_2_ = the weight from the previous weighing; *n* = the number of days from the previous weighing to the assessed weighing (*n* = 7).

#### Determination of Feed Consumption and Feed Conversion

Feed consumption was recorded daily for each group throughout the fattening period. The average feed consumption for each group was calculated for a certain period (1st–7th, 8–14th, 15th–21st, 22nd–28th, 29–35th d and 38–39th d to the day of slaughter). Subsequently, the average daily feed consumption (**ADFI**) was recalculated per broiler.

Feed conversion was calculated as the ratio of the weight of feed consumed during fattening and the increase in the chicken weight achieved during the observed fattening period.

$$feed\;conversion=\frac{ADFI}{ADG}$$

### Homogenization of Samples and Isolation of Total RNA of Target Genes

Samples of the pectoral muscle and caudal part of the cecum (20 mg weighted pieces) were immediately placed in RNA Later solution (Qiagen, UK) and stored at –70°C before the RNA purification and transcription as mentioned in Karaffová et al. (2017).

### Gene Expression Analysis - Real Time-qPCR

Seven animals per group were randomly selected to determine relative gene expression of IGF-2, PAX-7, MYF-5 (pectoral muscle) as well as IgA, MUC2 (cecum) and GAPDH (glyceraldehyde-3-phosphate dehydrogenase) for normalisation. The reference gene GAPDH was selected based on confirmed expression stability using the GeNorm.

Amplification and detection of transcripts were performed using the LightCycler 480 II instrument (Roche, Switzerland) and Maxima SYBR Green qPCR Master Mix (Thermo Scientific, Waltham, MA). Subsequent Real Time-qPCR to detect relative mRNA abundance was performed for 38 cycles under the following conditions: initial denaturation at 95°C for 2 min, subsequent denaturation at 95°C for 15 s, annealing (as listed in Table 3) and extension step for 2 min at 72°C. A melting curve from 50 to 95°C with readings at every 0.5°C was recorded for each individual RT-qPCR plate. Analysis was performed after every run to ensure a single amplified product for each reaction. All reactions for qPCR were done in duplicate. The primer sequences, annealing temperatures and times for each primer used for RT-qPCR are listed in Table 3. All primer sets allowed cDNA amplification efficiencies between 94% and 100%. It was confirmed that the efficiency of amplification for each target gene (including GAPDH) was essentially 100 % in the exponential phase of the reaction, where the quantification cycle (Cq) was calculated. The Cq values of the studied genes were normalized to an average Cq value of the reference gene (ΔCq), and the relative expression of each gene was calculated as 2–^ΔΔCq^.

**Table 3:** List of primers used for the gene mRNA quantification in broilers.

Primer	Sequence 5′–3′	Annealing temperature / time	References
IGF-2 Fw	CTCTGCTGGAAACCTACTGT	54°C/ 30s	Mudroňová et al., 2018
IGF-2 Rev	GAGTACTTGGCATGAGATGG		
MYF-5 Fw	CAGAGACTCCCCAAAGTGGAGAT	60°C /1 min	Xiao et al., 2017
MYF-5 Rev	GTCCCGGCAGGTGATAGTAGTTC		
PAX-7 Fw	AGGCTGACTTCTCCATCTCTCCT		Adhikari et al., 2019
PAX-7 Rev	TGTAACTGGTGGTGCTGTAGGTG		
IgA Fw	GTCACCGTCACCTGGACTACA	55°C/30s	Lammers et al., 2010
IgA Rev	ACCGATGGTCTCCTTCACATC		
MUC-2 Fw	GCTGATTGTCACTCACGCCTT	54°C/1 min	Smirnov et al., 2006
MUC-2 Rev	ATCTGCCTGAATCACAGGTGC		
GAPDH Fw	CCTGCATCTGCCCATTT	59°C /30 s	De Boever et al., 2008
GAPDH Rev	GGCACGCCATCACTATC		

### Testing Phagocyte Function

The assessment of active phagocytes' proportion (%) and their ability to engulf particles was carried out using a commercial IngoFlowEx Kit (Exbio Praha a.s., Czech Republic). The oxidative burst activity of phagocytes was tested by the Phagoburst test (Celonic, Heidelberg, Germany). Both tests were conducted following the manufacturer's guidelines, using freshly collected heparinised blood. The prepared samples were analysed on a BD FACSCanto flow cytometer (Becton Dickinson Biosciences, Qume Drive San Jose, CA) equipped with BD FACS Diva software. Granulocytes and monocytes were sorted using the FSC versus SSC dot plot. Any bacterial aggregates were removed from subsequent analysis by considering their minimal DNA content as shown in the red fluorescence histogram (FL-2). For both tests, the proportion of active phagocytes and the mean fluorescence intensity were assessed using the green fluorescence histogram (FL-1).

### Lymphocyte Phenotyping

To identify specific subsets of lymphocytes, mononuclear cells were extracted from 600 μL of heparinized blood, which was diluted at a 1:1 ratio with phosphate buffer saline (PBS; MP Biomedicals, France). This diluted blood was carefully layered over 2.5 ml of Lymphosep, Lymphocyte Separation Media (Biosera, Nuaillé, France). After centrifugation at 600 x g for 30 min, mononuclear cells were collected from the interface between the separation solution and the plasma. The collected cells underwent 2 rounds of washing with PBS through centrifugation at 250 x *g* for 5 min. Subsequently, the concentration of mononuclear cells was assessed following staining with Türck's solution in a Bürker chamber, and it was adjusted to 5.10^5^ cells in 50 μL.

The identification of specific lymphocyte subsets was performed by direct immunostaining using conjugated monoclonal mouse anti-chicken antibodies (Southern Biotech, Birmingham, AL) in subsequent combinations: CD45/CD3/IgM, CD4/CD8a and Bu-1/Monocytes/Macrophage. The used antibodies are specified in Table 4. The cells were exposed to the antibodies for 20 min in a dark environment at room temperature. Following antibody exposure, the cells were washed twice with 1 mL of PBS (at 250 x *g* for 5 min), resuspended in 100 μL of PBS and then analyzed on the same flow cytometer as described above. For subsequent analysis, lymphocytes and monocytes were gated on a baseline dot plot (FSC vs. SSC), and contaminating thrombocytes were distinguished from lymphocytes based on their higher granularity (Bertram, 1998).

**Table 4:** Specification and amounts of used mouse anti-chicken monoclonal antibodies.

Type	FL	Clone	Isotype	Concentration	Amount/5×10^5^ cells
anti-Bu-1	R-PE	AV20	IgG1, κ	0.1 mg/mL	0.5 μL
anti-CD3	FITC	CT-3	IgG1 κ	0.5 mg/mL	2 μL
anti-CD4	FITC	CT-4	IgG1, κ	0.5 mg/mL	2 μL
anti-CD8a	R-PE	CT-8	IgG1, κ	0.1 mg/mL	1 μL
anti-CD45	APC	LT-40	IgM, κ	0.1 mg/mL	5 μL
anti-IgM	R-PE	M-1	IgG2b κ	0.1 mg/mL	1 μL

Abbreviations: CD, cluster of differentiation; IgM, immunoglobulin M; MO, monocyte; MF, macrophage; FL, fluorochrome.

CD3+ lymphocytes represent T lymphocytes, IgM+ and Bu-1 cells are subsets of B lymphocytes. CD4+CD8a- and CD4+CD8a^low/mid^ subsets were added together and they represent T helper lymphocytes, while CD4-CD8a+ subset represent T cytotoxic cells (Figure S1: Gating strategy). Proportions of mononuclear cells are expressed in percentage.

### Statistical Analysis

Statistical analysis of gene expression data was done using one-way ANOVA with Tukey's post hoc analysis using Graph Pad Prism version 8.00. Differences between the mean values for different treatment groups were considered statistically significant at *P <* 0.0001. Values in figures are given as means with standard deviations (± SD).

Statistical analysis of production parameters and parameters of cellular immunity (phagocytosis and lymphocyte phenotypisation) was performed using GraphPad Prism Version 9.0.0 software (GraphPad Software, Inc., San Diego, CA). The Shapiro–Wilk test was used to analyze data normality. Since the data showed normality, they were further tested by one-way ANOVA followed by post hoc multicomparison Tukey's test to determine differences between the groups. Differences between groups were considered significant if the p-value was lower than 0.05. Data are expressed as means ± standard deviations.

## RESULTS

### Production Parameters

In this study, the effect of 0.6% addition of HS and probiotic strain *Limosilactobacillus fermentum* 2i3 on growth parameters and feeding rate of broilers was also investigated. In Table 5 we can observe the average weight of broilers during fattening. We noticed a statistically significant difference during fattening only in the 2nd wk, when the average weight was the highest in the control and probiotic groups compared to H and PH. In the last 2 wk of fattening, the highest weights were in the probiotic group, but without a statistically significant difference compared to the other groups.

**Table 5:** The influence of administration of humic substances, probiotics and their combination on the average body weight (g/1 pcs. ± standard deviation) of broiler chicken, ^a-b^ different letters indicate significant differences among all groups and time points at *P < 0.05*.

	C	H	P	PH	*P*
1st wk	209.7 ±12.0	194.5 ± 12.8	200.5 ± 17.0	202.5 ± 11.2	0.107
2nd wk	465.03 ± 14.3^a^	435.3 ± 10.7^b^	466.7 ± 29.5^a^	455.9 ± 11.5^ab^	0.013
3rd wk	905.8 ± 46.1	852.6 ± 41.6	882.6 ± 67.1	872.0 ± 78.0	0.257
4th wk	1491.4 ± 6.9	1449.0 ± 44.1	1499.0 ± 79.6	1444.0 ± 36.5	0.422
5st wk	2068.2 ± 109.6	2012.4 ± 51.4	2133.7 ± 168.5	2007.6 ± 46.5	0.136
Fattening	2158.2 ± 96.8	2232.0 ± 52.4	2306.8 ± 155.9	2197.0 ± 51.7	0.168

Table 6 presents the average weekly weight gains of broilers. Group P animals had the highest weekly weight gains in almost all measured observation periods (wk 2: 272.2 g; wk 4: 617.1 g; wk 5: 634.0 g) in comparison with other groups. Moreover, the animals in the probiotic group reached almost 150 g higher gain throughout the fattening period.

**Table 6:** The influence of administration of HS, probiotics and their combination on the average weekly weight gain (g/1 pcs.) of broiler chicken.

	C	H	P	PH
1st wk	168.7	159.8	154.4	161.6
2nd wk	255.3	234.8	272.2	253.4
3rd wk	440.8	417.3	415.9	416.9
4th wk	585.7	597.0	617.1	571.3
5th wk	576.8	562.9	634.0	563.5
Fattening	2117.2	2191.3	2266.0	2157.0

Table 7, Table 8 show the weekly and total feed consumption and feed conversion per kg of live weight, respectively. There is no statistically significant value for this parameter, but in the first half of the fattening period, the P group had the lowest feed consumption, and throughout the entire fattening period, the P group had the best feed conversion of 1.64 kg feed per 1 kg of live weight production. In the second part of the fattening period, the control group had the lowest feed consumption. However, the C group had the highest feed conversion of 1.76 kg.

**Table 7:** The influence of administration of HS, probiotics, and their combination on the average weekly feed consumption per broiler (g ± standard deviation).

	C	H	P	PH	*p*
1st wk	140.3 ± 10.2	142.8 ± 8.8	140.1 ± 8.5	141.8 ± 8.5	0.996
2nd wk	403.8 ± 28.6	385.4 ± 27.8	384.6 ± 25.3	395.0 ± 26.1	0.997
3rd wk	652.2 ± 13.6	646.2 ± 12.7	615.4 ± 12.2	645.0 ± 12.5	0.868
4th wk	866.2 ± 13.4	878.7 ± 12.3	835.3 ± 10.9	876.0 ± 13.0	0.779
5th wk	1,144.0 ± 13.0	1,158.0 ± 12.4	1,211.5 ± 25.3	1,186.0 ± 21.3	0.779
Fattening	3,790.5 ± 55.9	3,807.4 ± 58.0	3,794.9 ± 60.6	3,812.1 ± 57.7	1.000

**Table 8:** The influence of administration of HS, probiotics and their combination on the feed conversion ratio (feed consumption per 1 kg of live weight) of broilers.

	C	H	P	PH
1st wk	0.87	0.92	0.88	0.88
2nd wk	1.58	1.60	1.44	1.56
3rd wk	1.48	1.55	1.48	1.55
4th wk	1.48	1.47	1.35	1.54
5th wk	2.00	2.11	1.91	2.11
Fattening	1.76	1.71	1.64	1.73

### Relative Gene Expression of Selected Parameters

**Figure 1:**
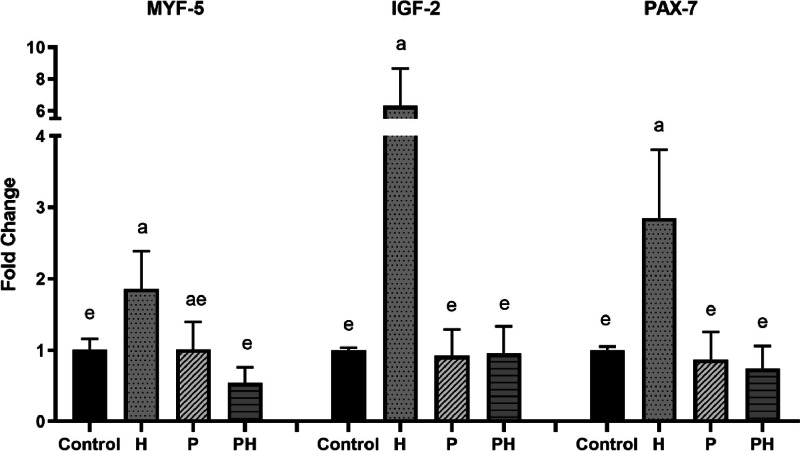
Gene expression of MYF-5, IGF-2 and PAX-7 in pectoralis muscles of broilers. Results at each time point are fold changes of expression of control. ^a-e^ different letters indicate significant differences among all groups and time points at *P < 0.0001*.

**Figure 2:**
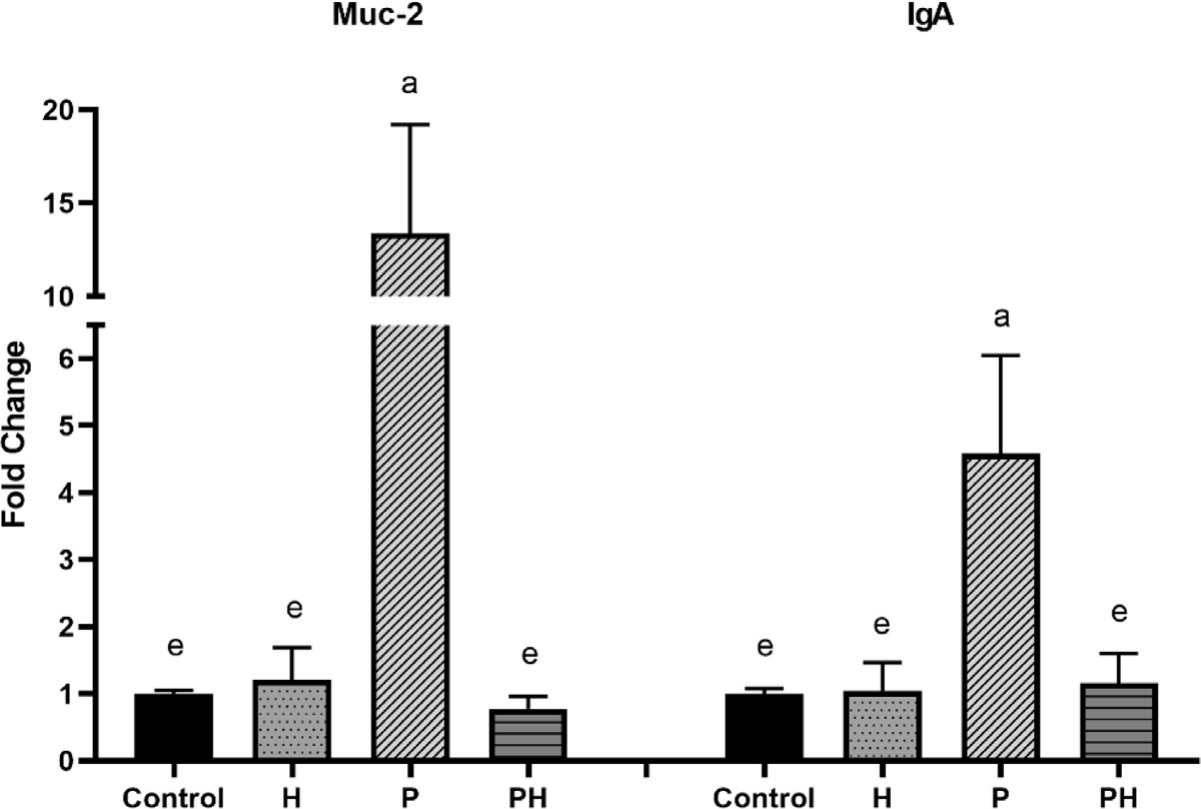
Gene expression of Muc-2 and IgA in cecum of broilers. Results at each time point are fold changes of expression of control. ^a-e^ different letters indicate significant differences among all groups and time points at *P < 0.0001*.

Gene expression, calculated as fold changes to control, is presented in Figure 1 (for myogenic regulatory and transcription factors) and Figure 2 (mucosal intestinal immunity). Relative gene expression for all factors was the highest in the humic group in comparison with other groups (*P <* 0.0001*)* (Figure 1). On the contrary, Muc-2 and IgA gene expression was markedly upregulated in the probiotic group alone compared to other groups (*P <* 0.0001*)* (Figure 2).

### Cellular Immune Response – Phagocyte Activity

**Figure 3:**
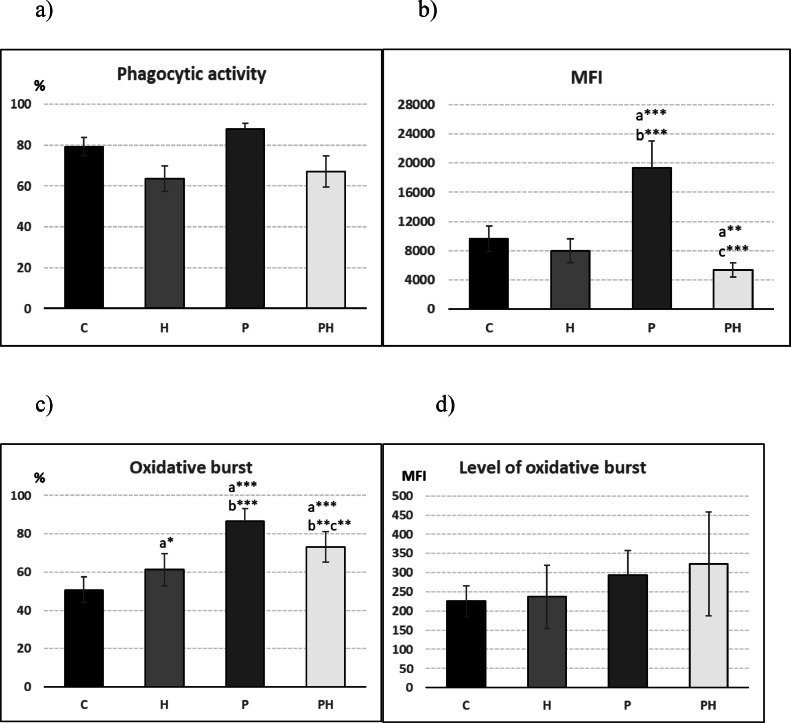
Influence of 0.6 % HS and *L. fermentum* 2I3 on activity of phagocytes in broiler blood evaluated as: (A) percentage of active phagocytes (phagocytic activity), (B) engulfing capacity of phagocytes (expressed as mean fluorescence intensity – MFI) (C) oxidative burst and (D) level of oxidative burst (n = 10). *A – significantly different from the control (C), B – significantly different from the group H, C – singnificantly different from the group P;* *P < 0.05; ^⁎⁎^*P* < 0.01; ^⁎⁎⁎^*P* < 0.001.

The effect of probiotic bacteria and/or HS on phagocytosis was monitored based on the percentage of actively phagocyting cells, their engulfing capacity, the percentage of phagocytes with oxidative burst and the level of oxidative burst. The addition of the probiotic strain *L. fermentum* 2i3 to the feed of broilers significantly increased both the percentage of active phagocytes and their absorption capacity compared to the control as well as to the groups where HS were applied. On the contrary, the addition of humic substances, either alone or in combination with probiotics, significantly reduced both mentioned parameters compared to the control (Figures 3A and 3B).

A significantly higher percentage of phagocytes with an oxidative burst was recorded in the probiotic group compared to all other groups. Likewise, in the groups fed HS alone or in combination with the probiotic, there was a significant increase in the percentage of cells in oxidative burst compared to the control (Figure 3C). The level of the oxidative burst was not significantly affected, only in the groups where the probiotic strain was applied (P and PH), a tendency to increase compared to the control could be observed (Figure 3D).

**Figure 4:**
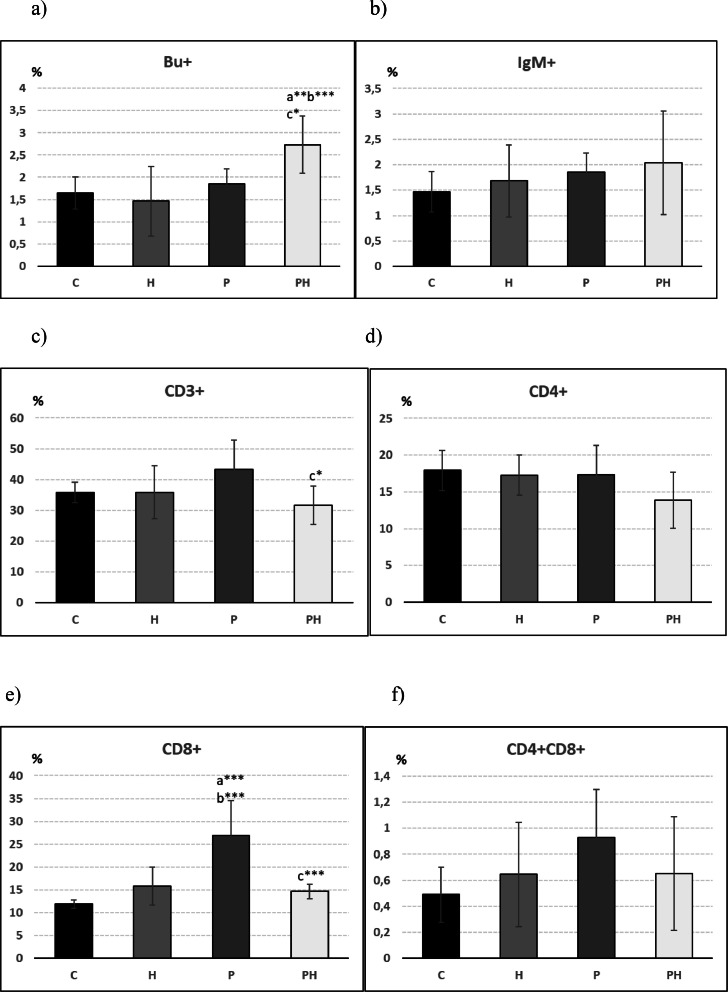
Percentage of (A) BU+, (B) IgM+, (C) CD3+, (D) CD4+, (E) CD8+, (F) CD4+CD8+ T lymphocytes in blood of broilers (n = 10). *A – significantly different from the control (C), B – significantly different from the group H, C – singnificantly different from the group P;* *P < 0.05; ^⁎⁎^*P* < 0.01; ^⁎⁎⁎^*P* < 0.001.

Within the monitoring the effect of HS and probiotics on lymphocyte subpopulations, it was found that the combination of HS and probiotics significantly increased the percentage representation of Bu-1+ B-lymphocytes. The same tendency was observed in the case of the IgM+ subpopulation of B-lymphocytes, but without statistical significance (Figures 4A and 4B). The increased representation of B-lymphocyte subpopulations after feeding the combination of HS and probiotics was reflected in a tendency to decrease the representation of total CD3+ T-lymphocytes as well as CD4+ T-lymphocytes in this group (Figures 4C and 4D). Statistically significant changes were noted in CD8+ and CD4+CD8+ T-lymphocyte subpopulations, where the administration of probiotic strain 2i3 alone caused a significant increase compared to the control (Figures 4E and 4F). In the groups of broilers fed HS (H and PH), the representation of T-lymphocyte subpopulations was not significantly different from the control (Figures 4C–4F).

## DISCUSSION

It is believed that probiotic bacteria, as well as HS, regularly show positive effects on health, but their direct impact on some production indicators, as well as transcriptional myogenic factors are often contradictory. Moreover, the use of antibiotics as growth promotors is prohibited in the European Union, therefore it is necessary to study these substances of natural origin in detail so that they can become an alternative solution in this regard. In addition, accelerated breast muscle growth is a desirable trait in poultry species raised for meat production.

However, skeletal muscle is known to exhibit a remarkable capacity for regeneration, a process that is dependent on satellite cells. Thus, the management of satellite cell activity is also crucial in terms of skeletal muscle development. In this sense, the protein IGF-2 and MYF-5 have a specific role in the regulation of skeletal muscle differentiation during myogenesis, as well as regeneration by coordinating satellite cell function (Zammit, 2017; Torrente et al., 2020). During embryonic skeletal muscle development, another transcription factor, PAX-7, plays a strategic role in the formation and differentiation of precursor cells. At the same time, it controls the expression of other myogenic regulators (Florkowska et al., 2020).

In our study, mainly the addition of 0.6% HS to the feed increased the relative expression of genes for all myogenic factors (MYF-5, IGF-2, PAX-7). On the contrary, in our previous study monitoring the effect of adding 0.8% HS to broiler feed, we recorded a significant decrease in gene expression for IGF-2 (Mudroňová et al., 2020). Based on the results obtained, we hypothesize that the concentration of the added HS may have a decisive impact on myogenesis. Moreover, the influence of different concentrations of HS or their combination with probiotic strains on myogenic transcription factors has not yet been studied in poultry.

On the other hand, the combination of the probiotic strain with HS did not produce the expected stimulation of breast muscle growth at the molecular level. Interestingly, even the administration of *L. fermentum* 2i3 alone did not have a sufficiently stimulating effect on the gene expression of these factors. Notably, in our recent study by Albrecht et al. 2022, we found that another probiotic strain, *Enterococcus faecium* AL41, had only a temporary stimulatory effect on IGF-1 gene expression, which declined as the chickens aged, and in contrast, had a strong effect on PAX-7 expression. These results may indicate different effects of individual probiotic strains at the molecular level of the growth factors in the pectoral muscle. Nevertheless, in the aforementioned study, there was an overall positive effect on pectoral muscle growth.

Our results showed that administration of *L. fermentum* 2i3 at a dose of 1 × 10^9^ CFU/mL of drinking water had a positive effect on some production parameters such as feed conversion ratio and weekly weight gain. Likewise, several recent studies have reported that commercial probiotic products in which the concentration of probiotic bacteria used is at least 1 × 10^8^ CFU/g of feed, demonstrably improve production parameters as well as the immune response of broiler chickens (Rehman et al., 2020; Fesseha et al., 2021; Zou et al., 2022). Most authors believe that the beneficial effects of probiotics can be increased by selecting “strong strains”, combining 2 or more strains, gene manipulation, or even the increasingly popular combination of probiotics and other synergistic substances of natural origin. However, in our experiment, the stimulating effect of the combination of *L. fermentum* 2i3 and 0.6% addition of HS on the selected production parameters of broilers was not confirmed. We hypothesize that this may be caused by a certain mutual antagonistic action, e.g. by absorbing growth elements of probiotic bacteria or even nutrients by HS.

Another positive effect of many substances of natural origin, including probiotics and HS, is their immunomodulating effect on the body, which has been noted by numerous authors in various animal species (Cetin et al., 2011; Wang et al., 2020; Mudroňová et al., 2021; Trofimova et al., 2021). In our previous studies, we also observed the stimulating effect of humic substances on the activity of phagocytes, the representation of some lymphocyte subpopulations in the blood, as well as on the expression of genes for IgA or mucin-2 in the intestinal tissue. We found that their effect depends on the concentration used, the duration of application and the category of poultry (Mudroňová et al., 2020; Mudroňová et al., 2021). In this experiment, we applied a new preparation with HS to broilers, where the manufacturers use a special technology for its preparation intended for the detoxification of feed mixtures from mycotoxins, bacterial toxins, heavy metals and other organic toxins. The manufacturers state that when natural leonardite is processed to a particle size of less than 200 µm, there is only minimal damage to the humic acid molecules, which are then able to bind the toxins mentioned (https://humac-farma.sk). In general, the molecular weights of HS range from hundreds of thousands to millions of daltons (Perminova et al., 2003). It can be assumed that the HUMAC Natur AFM MycotoxiSorb preparation used in this experiment will therefore also contain humic acids with a very high molecular weight. Surprisingly, when using this type of HS, we did not observe any stimulatory effect on the engulfing activity of phagocytes or the expression of genes for IgA and Muc-2, and only a minimal effect on the representation of lymphocyte subpopulations. Stimulation after the application of these HS occurred only in the case of an oxidative burst, where the percentage of activated phagocytes was significantly increased. The immunomodulating effect of HS is mainly explained by the formation of complexes with biogenic molecules (e.g. amino acids, peptides and sugars), which then bind to the surface of lymphocytes and influence the production of cytokines regulating other immune reactions. This pathway is relevant for low-molecular HS (Riede et al., 1991). Molecules of humic acids contain structural segments capable of stimulating various biological activities. Our hypothesis is that when humic acid molecules are excessively large, these segments may be shielded by other parts of the molecular structure. As a result, they become unavailable for interaction with other biogenic molecules or with receptors on immune cells. The second hypothesis is that these HS with high absorption capacity can also capture smaller cytokine molecules (5–25 kDa) within their structure, thereby mitigating their impact on immune reactions. In the following research, it will be necessary to verify the exact mechanism of action of HS, compare the abilities of HS with low and high molecular weight and also to test their ability to bind cytokines.

On the other hand, we observed the expected stimulation of phagocytosis after the administration of probiotics, where there was a significant increase in the engulfing capacity of phagocytes as well as the percentage of phagocytes in oxidative burst compared to the control. Likewise, there was also a statistically significant increase in the expression of IgA and Muc-2 genes in the intestinal tissue compared to the control, as well as the other experimental groups. Regarding the representation of lymphocyte subpopulations in the blood after the application of probiotics, there was a significant increase in the percentage of cytotoxic T lymphocytes and double-positive T lymphocytes compared to the other groups. The immunomodulating effect of probiotic LAB has been confirmed in many studies in poultry. Stimulation of phagocytosis, NK cells, antibody production, influence of cytokine production with subsequent regulation of the representation and activity of individual subpopulations of lymphocytes were recorded, both at the level of mucosal and total immune response (Koenen et al., 2004; Dalloul et al., 2005; Haghighi et al., 2005; Haghighi et al., 2008; Fathi et al., 2017).

Similar to the monitoring of production parameters or myogenic factors, we did not observe a positive effect of the combination of probiotics and HS on the immune response either. The results of most of the immune parameters we tested (Muc-2, IgA, phagocyte activity, T lymphocyte subpopulations and IgM+ B lymphocyte subpopulation) were almost identical in the combined group as in the group where only HS was administered. These results support our hypothesis, mentioned above, that HS with high molecular weight bind not only toxins or heavy metals, but also biogenic molecules (e.g., cytokines) or even bacteria themselves. The binding of bacteria to the structure of the humic substance – alginate – has been confirmed and is part of the patent no. 288782, which is registered at the Office of Industrial Property of the Slovak Republic (Styková et al., 2016).

## CONCLUSIONS

Based on our obtained findings, we may confirm the immunostimulating effect of *L. fermentum* 2i3 administered in drinking water, which also had an impact on important production parameters of broilers. The application of a new formula of 0.6% humic substances, specifically designed for detoxification, significantly increased the relative gene expression for all myogenic growth factors, but showed no effect on production parameters. Moreover, it had a negative effect on phagocytic activity. However, the combination of probiotic strain with humic substances did not significantly affect the monitored parameters. In addition, this study brings knowledge about the influence of the technology used to process humic substance on important production and immunological indicators, which can be used for specific purposes in poultry farms. Based on this and our previous studies mentioned in the Discussion, where we applied HS to poultry, we can conclude that their effect on the immune response depends not only on the dose and source used, but also on the processing technology, which affects the size and structure of the humic acid molecules. Very large humic acid molecules with high absorption capacity appear to have much lower immunostimulatory abilities compared to smaller molecules. To confirm this hypothesis, it will be necessary to accurately analyse the structure of the humic acid molecules in the used HS.

## DISCLOSURES

The authors declare no conflict of interest.

## Appendix Supplementary materials

Image, application 1

## Footnotes

Supplementary material associated with this article can be found in the online version at doi:10.1016/j.psj.2024.103884.
