# Supplementary material for: The effect of Limosilactobacillus fermentum 2i3 and 0.6% addition of humic substances on production parameters and the immune system of broilers
Source: Poult Sci. 2024 May 22;103(8):103884. doi: 10.1016/j.psj.2024.103884 (PMC11223114; doi:10.1016/j.psj.2024.103884)
Supplement: Supplementary file 1 [file mmc1.docx]

1. B)


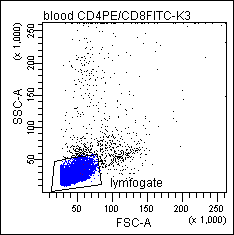

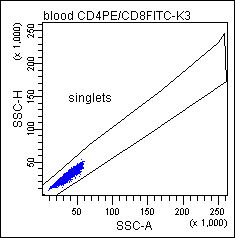


1. D)


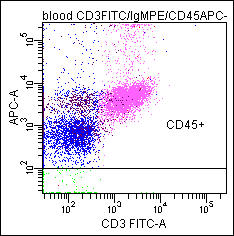

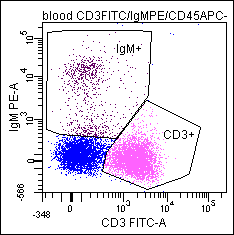


E)


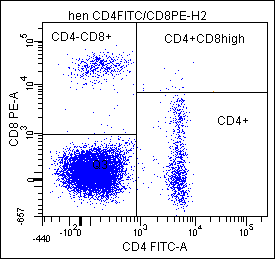


Figure S1: Phenotypization of lymphocytes – gating:

1. Statement of the position of lymphocytes on dot plot FSC-A versus SSC-A;
2. Selection of single cells on dot plot SSC-A vs SSC-H;
3. Gating of CD45+ lymphocytes;
4. Gating of CD3+ and IgM+ lymphocytes;
5. Gating of T lymphocytes subpopulations: CD4+ subpopulation including CD4+CD8^low/mid^ lymphocytes representing T helper and regulatory lymphocytes; CD4-CD8+ represents T cytotoxic cells
